# Conference 2022: Let's try this again…

**DOI:** 10.29173/jchla29614

**Published:** 2022-04-01

**Authors:** Thomas Blennerhassett

The Conference Planning Committee has been hard at work putting together our **hybrid conference** in Niagara Falls, Ontario!

Yes, to enable ALL our members to have access to this year’s presentations and content, we will have a conference that is in-person and with a virtual attendance option! We know from the very successful virtual conference last year in Winnipeg that virtual is an option that encourages participation from our members far and wide.

This year we will have several exciting CE classes, as well as virtual and in-person social events in the beautiful Niagara Falls region. Perfect for connecting with colleagues that we have been distant from over the past several years. Virtual trivia nights, dine-around options, and a banquet are all on the schedule for the big weekend.

As we were almost in Niagara Falls in 2020, so our theme this year is: Let's try this again… / Essayons encore une fois…

Mark your calendars now! Saturday, June 11 – Monday, June 13, 2022.

See you in Niagara Falls – in person or online! Find more information on our conference website.

**Congrès 2022: Essayons encore une fois… /Let's try this again…!**

Le comité de planification de la conférence a travaillé d'arrache-pied pour organiser notre **congrès hybride** à Niagara Falls, en Ontario.

Oui, afin de permettre à TOUS nos membres d'avoir accès aux présentations et au contenu de cette année, nous aurons une conférence en présentiel et une option virtuelle. Grâce à la conférence virtuelle très réussie de l'année dernière à Winnipeg, nous savons que l’option virtuelle encourage la participation de nos membres de partout à travers le pays.

Cette année, nous aurons plusieurs formations continues passionnantes, ainsi que des événements sociaux virtuels et en présentiel, dans la magnifique région de Niagara Falls. C'est l'occasion idéale de retrouver des collègues, dont nous nous sommes éloignés au cours des dernières années. Des soirées trivia virtuelles, des options de restauration et un banquet sont tous au programme de ce grand weekend.

Comme nous avons failli nous croiser à Niagara Falls en 2020, notre thème cette année est: Essayons encore une fois… / Let's try this again…

Inscrivez ces dates à vos calendriers! Du samedi 11 juin au lundi 13 juin 2022.

Rendez-vous à Niagara Falls - en personne ou en ligne!

Vous trouverez plus d’informations sur le site Web du congrès.

